# Predicting pneumonia during hospitalization in flail chest patients using machine learning approaches

**DOI:** 10.3389/fsurg.2022.1060691

**Published:** 2023-01-06

**Authors:** Xiaolin Song, Hui Li, Qingsong Chen, Tao Zhang, Guangbin Huang, Lingyun Zou, Dingyuan Du

**Affiliations:** ^1^ School of Medicine, Chongqing University, Chongqing, China; ^2^ Department of Traumatology, Chongqing Emergency Medical Center, Chongqing University Central Hospital, Chongqing, China; ^3^ Clinical Data Research Center, Chongqing Emergency Medical Center, Chongqing University Central Hospital, Chongqing, China

**Keywords:** flail chest, pneumonia, machine learning, risk factors, extreme gradient boosting

## Abstract

**Objective:**

Pneumonia is a common pulmonary complication of flail chest, causing high morbidity and mortality rates in affected patients. The existing methods for identifying pneumonia have low accuracy, and their use may delay antimicrobial therapy. However, machine learning can be combined with electronic medical record systems to identify information and assist in quick clinical decision-making. Our study aimed to develop a novel machine-learning model to predict pneumonia risk in flail chest patients.

**Methods:**

From January 2011 to December 2021, the electronic medical records of 169 adult patients with flail chest at a tertiary teaching hospital in an urban level I Trauma Centre in Chongqing were retrospectively analysed. Then, the patients were randomly divided into training and test sets at a ratio of 7:3. Using the Fisher score, the best subset of variables was chosen. The performance of the seven models was evaluated by computing the area under the receiver operating characteristic curve (AUC). The output of the XGBoost model was shown using the Shapley Additive exPlanation (SHAP) method.

**Results:**

Of 802 multiple rib fracture patients, 169 flail chest patients were eventually included, and 86 (50.80%) were diagnosed with pneumonia. The XGBoost model performed the best among all seven machine-learning models. The AUC of the XGBoost model was 0.895 (sensitivity: 84.3%; specificity: 80.0%).

Pneumonia in flail chest patients was associated with several features: systolic blood pressure, pH value, blood transfusion, and ISS.

**Conclusion:**

Our study demonstrated that the XGBoost model with 32 variables had high reliability in assessing risk indicators of pneumonia in flail chest patients. The SHAP method can identify vital pneumonia risk factors, making the XGBoost model's output clinically meaningful.

## Introduction

Flail chest is the most severe type of chest trauma, is found in approximately 4% of patients with rib fractures and is defined as at least 3 or 4 consecutive rib fractures in at least two places causing paradoxical movement of the chest wall (1, 2). It has been described in 30% of patients with significant chest trauma requiring intensive care, and flail chest has an adverse impact on their respiratory function and causes several associated complications, such as pneumonia and acute respiratory distress syndrome (ARDS) (3).

Pneumonia is a common, severe, preventable complication in patients with flail chest, with an estimated incidence rate ranging from 21% to 33.5% (4–6). Moreover, pneumonia morbidity is an important indicator of care quality for patients with rib fractures, including risks of prolonged mechanical ventilation, increased mortality, and poor long-term outcomes (5). There are some challenges in managing pneumonia (7). Overestimating the likelihood of pneumonia can cause inappropriate use of antibiotics, leading to the emergence of multidrug-resistant and invasive fungal infections. Conversely, underestimating the likelihood of pneumonia leads to undertreatment and increased mortality from severe nosocomial infections (8, 9). Thus, to avoid possible adverse disease progression and unfavourable outcomes, it is essential that physicians accurately identify patients at high risk of pneumonia at an early stage and tailor individualised preventive treatment (e.g., enhanced monitoring, lung hygiene, pain management) (10).

The Clinical Pulmonary Infection Score (CPIS) has been proposed and used clinically for decades and has shown some shortcomings in guiding the management of pneumonia (8). Consequently, many studies have been conducted to design and construct more accurate and stable risk scores, such as the Scoring System for Pneumonia Risk in Pulmonary Contusion Patients (11) and “RibScore”, a novel radiographic score based on fracture patterns, have been performed (12). These prediction models have been developed based on a generalised linear approach; in addition, the “RibScore” only considers anatomical factors (13). However, many risk factors may exhibit a nonlinear relationship with the outcome and may not fully apply to the traditional linear regression prediction model (14).

Machine learning (ML), a branch of artificial intelligence methods, can develop models from medical data to make clinical decisions and assist doctors in their routine work (15). In previous studies, several classical machine learning algorithms have been tested to predict the risk of pneumonia, including the prediction of stroke-associated pneumonia (16), ventilator-associated pneumonia (17), and postoperative pneumonia (18). However, studies in which researchers use machine learning to predict pneumonia risk in flail chest patients are rare. Even though these algorithms have not yet become widely accepted and used in clinical decision-making, they have tremendous potential in medicine (19).

In this study, we aimed to develop an advanced machine learning model, assess how well it predicts the risk of pneumonia in flail chest patients, and provide new approaches for individualized analyses of pneumonia risk factors in hospitalized patients with flail chest.

## Materials and methods

### Study design and participants

**Figure 1:**
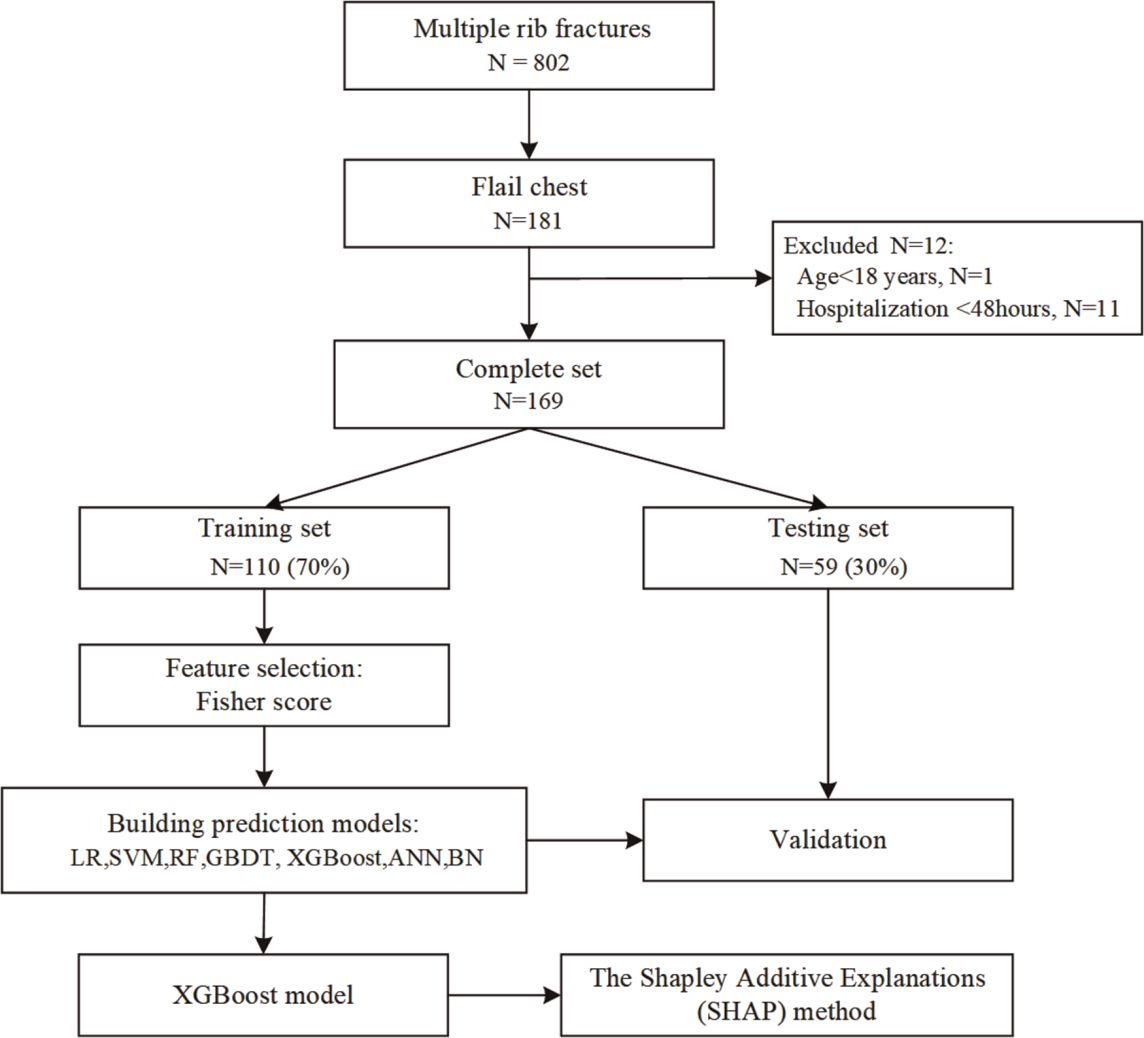
Flowchart for patient selection and dataset partitioning. LR, logistic regression; SVM, support vector machine; GBDT, gradient boosting decision tree; XGBoost, extreme gradient boosting; ANN, artificial neural network; NB, naive Bayes.

We performed a retrospective cohort study at Chongqing Emergency Medical Center, an urban teaching hospital with 1,200 beds in China, between January 2011 and December 2021. The study included patients with flail chest, defined as at least 3 or 4 consecutive rib fractures in at least two places causing paradoxical movement of the chest wall (2). We excluded patients <18 years of age, patients who died within 48 h of admission or had pneumonia before referral to our institution, and patients with rib fractures due to cardiopulmonary resuscitation (Figure 1).

### Primary outcome

Pneumonia status was determined by a combination of medical record documentation and data logger verification. Pneumonia was defined as the presence of new progressive infiltrates with at least two of the following symptoms: purulent respiratory secretion; body temperature ≥38°C or ≤35°C; leukocytosis (white blood cell count of ≥10,000/mm^3^) or leucopoenia (white blood cell count of ≤4,500/mm^3^, or immature neutrophils exceeding 15%) (5).

### Data collection

Data were collected from the electronic medical records:

(1) Demographics: age, sex, injury mechanism; (2) Damage level: Injury Severity Score (ISS), number of rib fractures, combined injuries, Glasgow Coma Scale (GCS), admission Revised Trauma Score (RTS), abbreviated injury scale (AIS); (3) Initial vitals: admission temperature, pulse rate, breathing rate, blood pressure; (4) Laboratory values in the first 48 h: blood gas analysis, WBC, RBC, HB, PLT, PCT, CRP, PT, APTT, INR, albumin; (5) Treatment within the first 48 h: transfer, intubation, emergency operation, closed thoracic drainage, tracheostomy, antibiotics, anticoagulants, emergency room stay time, etc.

### Variable selection

Feature selection is an indispensable preprocessing step for effectively analysing high-dimensional data (20). In our study, we first removed features that had missing values in more than 20% of the samples and then interpolated the remaining missing values using the k-nearest neighbour algorithm (18) (Impute Missing Values. KNN, in the Gene Pattern software package, http://www.broad.mit.edu/genepattern/). Second, the dataset with 169 records and 59 features was normalized using the Python Sklearn library. Then, the scores of each feature were computed and ranked based on the Fisher score evaluation system (21). Finally, we put features into model training, recursively deleted unimportant parts, and selected the optimal subset of each model.

The relevant model parameters were identified and reported for the best-performing prediction model.

### Statistical analysis

Demographic and clinical characteristics were analysed using descriptive statistics. Continuous variables that matched a normal distribution were expressed as the means ± SDs (standard deviations), and differences between pneumonia and nonpneumonia groups were analysed by t test or one-way ANOVA. Data that do not conform to a normal distribution are expressed as median (Interquartile range, IQR) and can be considered for logarithmic transformation. The F test was performed on the transformed data, and if normality was not achieved, the nonparametric test was applied. Categorical variables are presented as frequencies (percentages). Moreover, Fisher's exact test or chi-square test was used to compare the differences between the distributions of the two groups. *P *< 0.05 was considered statistically significant.

### Development of machine learning models

The following seven machine learning models with different algorithms were developed and evaluated for their performance: logistic regression (LR) (22), random forest (RF) (22), support vector machine (SVM) (23), gradient boosting decision tree (GBDT) (24), backpropagation artificial neural network (ANN) (23), extreme gradient boosting (XGBoost) (25), and naive Bayes (NB) (26). These algorithms have previously been shown to be stable and suitable for clinical datasets (27). The XGBoost model was built using the xgboost package, and the scikit-learn package constructed the remaining six models.

The 169 adult patients were then randomly divided into the training and validation sets: 70% (*n* = 118) into the training set and 30% (*n* = 51) into the validation set. We performed 3-fold cross-validation and grid search on the training set to tune the model hyperparameters and avoid overfitting. The training set was randomly divided into three subsets; in each iteration, one subset was selected as the test set, and the rest was chosen as the training set (24).

### Model evaluation

The hyperparameter set of each model was selected with the best model performance and was evaluated by the area under the receiver-operating curve (AUC), Matthews correlation coefficient (MCC), accuracy, sensitivity, specificity, positive predictive value (PPV), and negative predictive value (NPV). The best predictive model among the seven models was further used to analyse the risk factor gradient for flail chest pneumonia. Then, the calibration curve was used to evaluate the difference between the predicted and actual values.

### Model interpretation

The Shapley Additive Explanations (SHAP) is a model interpretability method widely used to interpret various classification and regression models (24, 28, 29). In this way, we can rank features according to their contribution to the model and visualize the association between features and outcomes (29). The model generated a predicted value for each sample, and the SHAP value represents the value assigned to each feature in the sample (29). Its absolute value reflects the impact of the feature, and its positive or negative value reflects its positive or negative effect on the prediction of the risk of developing pneumonia. When the SHAP value is >0, this indicates that the feature contributes to the risk of developing pneumonia; in contrast, when the SHAP value is <0, this indicates that the feature contributes less to the risk of developing pneumonia (29). The models were developed, evaluated, and interpreted using Python 3.6.1.

## Results

### Patient characteristics

During the study period, 802 patients with multiple rib fractures who were registered in our hospital were selected, and 633 (78.9%) patients were excluded. The search process and full inclusion/exclusion criteria are shown in Figure 1. Ultimately, 169 flail chest patients were recruited and used to develop and evaluate the machine learning models. Of these, 86 (50.8%) patients with pneumonia were roughly equally represented in the training set (50.8%) and the test set (51.0%). The median age of the patients was 56 years, 81.7% were male, and more than three-quarters were injured in traffic accidents (Table 1).

**Table 1:** Baseline characteristics of patients by clinical outcomes

Variables	No pneumonia (*n* = 83)	Pneumonia (*n* = 86)	*P-*value	Missing (%)
Demographic characteristics				
Age, Median (IQR)	54.5 ± 12.1	57.3 ± 15.0	0.186	0
Male, *n* (%)	66 (79.5)	72 (83.7)	0.612	0
Injury mechanism, *n* (%)			0.071	
Traffic accident	58 (69.9)	70 (81.4)		0
Fall from height	12 (14.5)	4 (4.7)		0
Fall from same level	4 (4.8)	1 (1.2)		0
Crush injury	9 (10.8)	11 (12.8)		0
Rib location, *n* (%)			0.004	
Left	31 (37.3)	17 (19.8)		0
Right	17 (20.5)	11 (12.8)		0
Bilateral	35 (42.2)	58 (67.4)		0
Rib fracture number, Median (IQR)	8.5 (6.0, 11.0)	10.0 (7.8, 15.0)	0.002	1.78
ISS, Mean ± SD	21.5 ± 7.9	31.2 ± 10.4	<0.001	0
GCS, Mean ± SD	14.5 ± 1.7	12.4 ± 3.5	<0.001	0
RTS, Mean ± SD	7.7 ± 0.6	7.0 ± 1.2	<0.001	0
Head AIS, *n* (%)			<0.001	
<3	72 (86.7)	52 (60.5)		0
≥3	11 (13.3)	34 (39.5)		0
Abdomen AIS, *n* (%)			0.038	
<3	76 (91.6)	68 (79.1)		0
≥3	7 (8.4)	18 (20.9)		0
Limbs and Pelvis AIS, *n* (%)			0.002	
<3	77 (92.8)	63 (73.3)		0
≥3	6 (7.2)	23 (26.7)		0
Payment method, *n* (%)			0.077	
Medicare payment	20 (24.1)	13 (15.1)		0
Self-pay	29 (34.9)	30 (34.9)		0
Employment injury insurance	20 (24.1)	15 (17.4)		0
Third party payment	14 (16.9)	28 (32.6)		0
Chest Comorbidities				
Pneumothorax, *n* (%)	57 (68.7)	62 (72.1)	0.75	0
Haemothorax, *n* (%)	47 (56.6)	60 (69.8)	0.107	0
Lung contusion, *n* (%)	68 (81.9)	77 (89.5)	0.232	0
Clavi-fracture, *n* (%)	14 (16.9)	18 (20.9)	0.633	0
Sternal fracture, *n* (%)	20 (24.1)	27 (31.4)	0.375	0
Thoracic vertebral fracture, *n* (%)	21 (25.3)	26 (30.2)	0.587	0
Vital signs				
T (°C), Mean ± SD	36.6 ± 0.4	36.7 ± 0.6	0.166	0
*P* (beats/min), Median (IQR)	84.0 (77.0, 97.0)	105.0 (88.0, 123.0)	<0.001	0
SBP (mm Hg), Mean ± SD	128.7 ± 22.0	117.1 ± 25.0	0.002	0
DBP (mm Hg), Mean ± SD	80.5 ± 13.1	71.5 ± 16.8	<0.001	0
Laboratory data in first 48 h				
Haemoglobin (g/L), Median (IQR)	123.0 (110.0, 137.5)	100.0 (82.5, 121.8)	<0.001	0
Platelets (10^9^/L), Median (IQR)	168.0 (138.5, 209.5)	151.5 (103.2, 203.0)	0.123	0
CRP transformation, Mean ± SD	0.9 ± 0.9	1.3 ± 0.9	0.02	7.69
White blood cell (10^9^/L), Median (IQR)	12.3 (9.0, 16.8)	13.2 (9.4, 18.1)	0.171	1.18
PT (second), Median (IQR)	13.6 (13.0, 14.4)	14.9 (13.7, 16.8)	<0.001	0
APTT (second), Median (IQR)	34.3 (31.4, 37.5)	36.7 (33.5, 42.0)	0.003	0
Albumin (g/L), Median (IQR)	39.0 (36.4, 42.2)	33.5 (28.1, 37.6)	<0.001	0
PH, Median (IQR)	7.4 (7.4, 7.4)	7.4 (7.3, 7.4)	0.145	17.75
PaO2 (mm Hg), Mean ± SD	105.8 ± 52.3	108.7 ± 55.9	0.754	17.75
PaCO2 (mm Hg), Mean ± SD	38.1 ± 6.7	36.6 ± 8.0	0.252	17.75
Oxygen saturation, Median (IQR)	98.1 (95.4, 99.0)	98.0 (94.7, 99.0)	0.533	17.75
Treatment in first 48 h				
Hospital transfer, *n* (%)	47 (56.6)	64 (74.4)	0.023	0
Emergency in ICU, *n* (%)	11 (13.3)	51 (59.3)	<0.001	0
Emergency intubation, *n* (%)	11 (13.3)	48 (55.8)	<0.001	0
Emergency close drain, *n* (%)	38 (45.8)	52 (60.5)	0.079	0
Emergency operation, *n* (%)	35 (42.2)	46 (53.5)	0.187	0
Tracheotomy, *n* (%)	3 (3.6)	35 (40.7)	<0.001	0
Blood transfusion (u), Median (IQR)	0.0 (0.0, 2.0)	4.0 (1.6, 9.4)	<0.001	0

Table 1 shows a summary of the demographic characteristics, laboratory findings, and clinical features of the patients enrolled with and without pneumonia. The demographic characteristics and comorbidities did not differ significantly between the patients with or without pneumonia (*P* > 0.05). Of note, respiratory rate, blood pressure, pulse, haemoglobin, c-reactive protein (CRP), prothrombin time (PT), activated partial thromboplastin time (APTT), and albumin were found to have significant differences between the patients with or without pneumonia (*P *< 0.05). In particular, the flail chest patients with pneumonia had higher ISS scores and more severe traumatic brain injury and were more likely to be intubated and transferred to the ICU at an early stage early (*P *< 0.05).

### Feature subset for prediction

We performed feature selection and ranked the levels of feature importance since only partially relevant or less significant characteristics are likely to have detrimental impact on the performance of machine learning models. The optimal feature subset for different machine learning algorithms may vary. Supplementary Figure S1 shows the Fisher score values in descending order, from which we selected the optimal subset of features for the seven models. The ideal LR model contains the top 14 features, the ANN model includes the top 19 features, and the remaining five models have the top 32 features.

### Prediction performance

**Figure 2:**
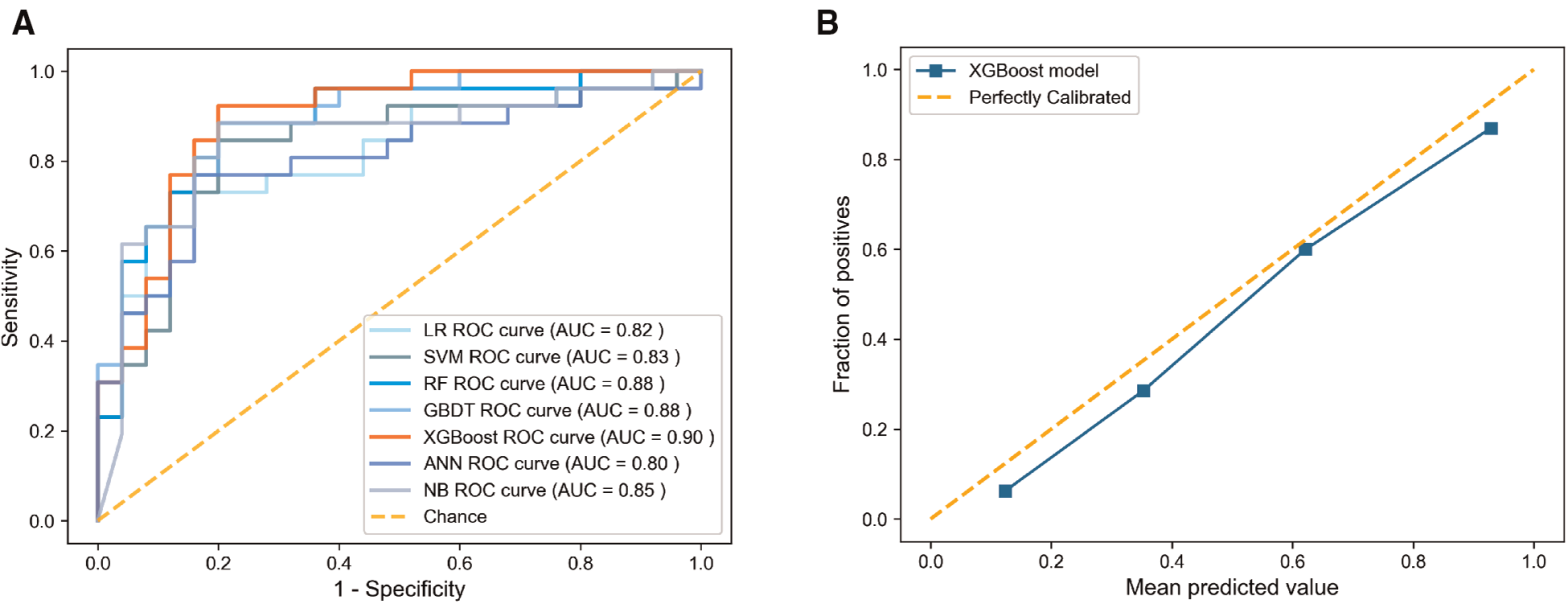
Discrimination and calibration performance of models. (**A**) ROC curves for the LR, SVM, RF, GBDT, XGBoost, ANN, and BN models in predicting pneumonia of flail chest, (**B**) Calibration curves for the XGBoost model. ROC, receiver operating characteristics; LR, logistic regression; SVM, support vector machine; GBDT, gradient boosting decision tree; XGBoost, extreme gradient boosting; ANN, artificial neural network; NB, naive Bayes.

The AUC, specificity, sensitivity, positive predictive value, negative predictive value, Matthews correlation coefficient (MCC), and accuracy of each model on the testing set are shown in Table 2 and Figure 2A. The AUC varied between 0.805 and 0.895 for different pneumonia prediction models: LR (0.818); SVM (0.831); RF (0.885), GBDT (0.880), ANN (0.805), XGBoost (0.895), and NB (0.848). The RF, NB, and XGBoost models all performed better than 80% in all metrics when using a probability cut-off value of 0.5, while the sensitivity of XGBoost (0.885) was much higher than that of RF and BN (0.808). The MCC of 0.688 and accuracy of 0.843 with XGBoost were also relatively high. Since the AUC values of the seven ML models did not differ much, we also considered the differences in other indicators, especially the sensitivity and MCC, and selected the XGBoost model as the final prediction model. The calibration curve for the XGBoost model in the test set is close to the 45 lines, which suggests that the model's predicted probability was close to the observed probability (Figure 2B). The hyperparameters of the XGBoost model were as follows: n estimators 250, learning rate 0.08, column sample by tree 0.8, minimum child weight 1, gamma 0.1, maximum depth 2, and subsample 0.72.

**Table 2:** Prediction performance of the machine learning models in the test set.

	**AUC**	Accuracy	Sensitivity	Specificity	**PPV**	**NPV**	**MCC**
**LR**	0.818	0.765	0.731	0.800	0.792	0.741	0.532
**SVM**	0.831	0.824	0.846	0.800	0.815	0.833	0.647
**RF**	0.885	0.804	0.808	0.800	0.808	0.800	0.608
**GBDT**	0.880	0.824	0.846	0.800	0.815	0.833	0.647
**XGBoost**	**0**.**895**	**0**.**843**	**0**.**885**	**0**.**800**	**0**.**821**	**0**.**870**	**0**.**688**
**ANN**	0.805	0.804	0.769	0.840	0.833	0.778	0.610
NB	0.848	0.824	0.808	0.840	0.840	0.808	0.648

LR, logistic regression; SVM, support vector machine; GBDT, gradient boosting decision tree; XGBoost, extreme gradient boosting; ANN, artificial neural network; NB, Naive Bayes; AUC, area under curve; PPV, positive predictive value; NPV, negative predictive value; MCC, Matthews correlation coefficient.

### Interpretability of the prediction model

**Figure 3:**
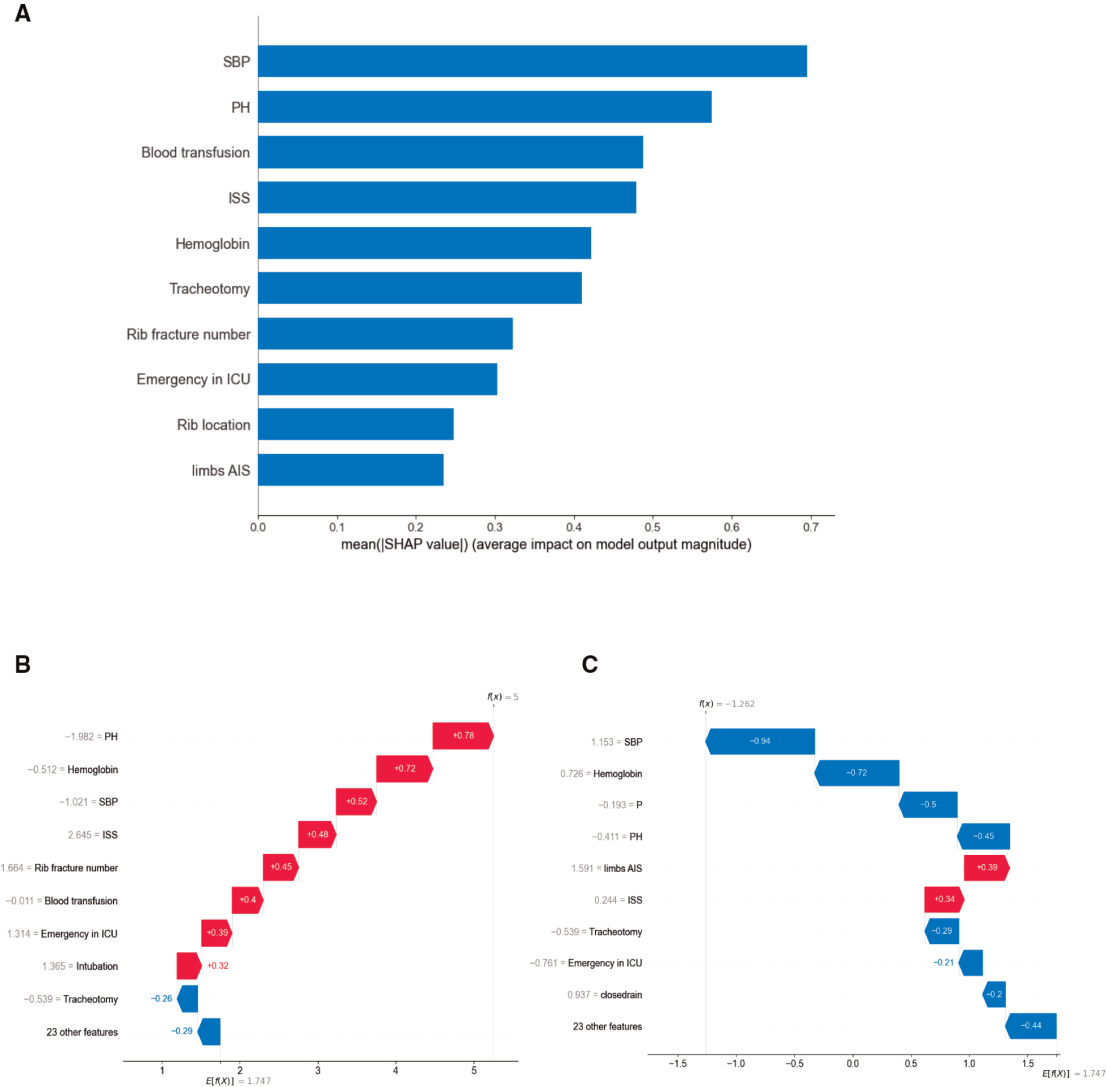
(**A**) the top 10 most important features indicated by XGBoost. (**B**) An illustrative example of the SHAP algorithm for interpreting the developed model. The bars in red and blue represent risk factors and protective factors, respectively; longer bars indicate greater feature importance. A patient with pneumonia accurately predicts the occurrence of pneumonia. (**C**) a patient without lung infection was accurately predicted to not suffer from pneumonia. PH, pH value; SBP, systolic blood pressure; ISS, Injury Severity Score; *P*, pulse.

To better explain the predictive meaning of the XGBoost model to guide clinical practice, we applied the SHAP algorithm to explain how to obtain the predicted probability based on the baseline risk and patient characteristics. As seen in Figure 3A, the importance of features is ranked according to the sum of the mean absolute value of all sample SHAP values. The top 10 important variables in the pneumonia prediction model were systolic blood pressure, pH value, blood transfusion, ISS, haemoglobin, tracheotomy, rib fracture number, emergency in ICU, rib location, and limbs or pelvis AIS.

Additionally, Figures 3B,C show the prediction results for a particular instance. Risk factors are shown in red and protective factors in blue; longer bars indicate greater importance of features. Figure 3B accurately predicts the occurrence of pneumonia in patients with pneumonia. Poor circulatory status with acidosis, requiring blood transfusion, and ICU treatment were risk factors for this patient. Figure 3C shows that a patient without lung infection was accurately predicted not to suffer from pneumonia. The XGBoost model can distinguish well between pneumonia and nonpneumonia patients and indicate different risk probabilities depending on the individuality of each hospitalized patient.

## Discussion

As a severe chest trauma, flail chest not only affects the stability of the chest wall but also poses a threat to circulation and breathing, which can directly affect the clinical course and outcome of the patient (30). Furthermore, multiple rib fractures and pulmonary contusions are significant trauma factors contributing to the development of pneumonia (31). Therefore, early detection of pneumonia is critical for timely interventions to improve clinical outcomes and reduce costs (32). In our study, we evaluated the performance of seven models for the occurrence of pneumonia in flail chest using clinical data from the first 48 h. The XGBoost model with 32 clinical features showed the best prediction ability, and we had some plans to avoid overfitting, such as feature selection by Fisher score and 3-fold cross-validation in the training set.

Predictive modelling to aid clinical decision-making is not a new concept. The Scoring System for Pneumonia Risk in Pulmonary Contusion Patients, the “RibScore”, a novel radiographic score based on fracture patterns, and the Clinical Pulmonary Infection Score (CPIS) are all examples of current operational scoring tools (8, 11, 12). The CPIS tool has shown moderate reliability and accuracy in diagnosing nosocomial pneumonia (33, 34). The CPIS, however, is based on radiographic and laboratory values following symptom onset, as with other tools (22). Thus, CPIS may assist clinicians in narrowing down the identification of patients with pneumonia, but treatment becomes delayed rather than early prevention.

Artificial intelligence and electronic medical records have enabled ML algorithms to be more widely used in individualized medicine to support clinical decisions (35). XGBoost models have shown advantages in predicting pneumonia due to their impressive predictive accuracy and ease of use (14). Chen et al. (36). based on the XGBoost algorithm, developed a risk prediction model for postoperative pneumonia in patients after liver transplantation. According to Li (37), XGBoost was used to build a machine learning model for predicting stroke-associated pneumonia with a 0.84 model evaluation precision. In our study, the XGBoost model has a sensitivity of 88.5%. In contrast, the specificity is 80.0%, meaning that only 11.5% of actual patients are not identified as high-risk (false-negative rate), yet 20% of the patients who do not have pneumonia are incorrectly labelled as high-risk patients (false-positive rate). This level of exposure to unnecessary interventions may be acceptable for a safe, inexpensive, and well-tolerated intervention (e.g., enhanced surveillance or pulmonary hygiene protocols) to prevent pneumonia in high-risk patients. In contrast, high sensitivity is essential in clinical practice because of the consequences of misclassification of actual patients with pneumonia. We can combine our models’ and doctors’ opinions to further improve system performance to overcome the effects of false negatives and positives.

Given these results, it appears that not all low-risk-level patients are pneumonia free. We report low-risk groups in several patients with pneumonia (Supplementary Table S1). This result reflects the incomplete clinical presentations initially observed in some patients. In the early admission stage, false-negative patients with clear consciousness and stable vital signs did not require emergency surgery or transfusion therapy, and the model identified them as a low pneumonia risk group. However, all three patients had injuries other than chest trauma, such as lower limb fractures and abdominal organ injuries, and their subsequent treatment and long-term immobilization increased the risk of pneumonia. On the other hand, one false-positive patient who was severely injured was predicted to have a high risk of pneumonia by the XGBoost model. The patient died during the early stages of hospitalization, and the development of pneumonia may not be effectively observed during the disease. Importantly, for patients with incorrect predictions in this model, we can incorporate dynamic clinical features (e.g., 24 and 48 h of admission) into the model for multiple predictions. Three or more clinicians will also combine their clinical experience to determine the likelihood of pneumonia in patients with flail chest (38). Clinicians could identify them effectively by incorporating clinical knowledge.

In addition, a predictive model for a disease needs to be interpretable, and this interpretability can promote understanding and acceptance of the model's predictions by physicians (28). Therefore, we used SHAP values to estimate how important predictor variables were to the model to gain insight into model interpretability. By taking the average of the absolute value of each feature's SHAP value and observing the importance to the overall model, we found that the more crucial baseline variables in the pneumonia prediction model were systolic blood pressure, pH value, blood transfusion, and Injury Severity Score (ISS). The first three indicators reflect the internal environment (39). The Injury Severity Score (ISS) is a common anatomical-based trauma score that can effectively assess the injury's severity and predict clinical outcomes such as the likelihood of survival and ICU length of stay (40, 41). However, it depends exclusively on anatomical factors of injuries, not synthesizing the mechanism of injury and physiological factors (42).

ML models have demonstrated outstanding performance in predicting diseases and clinical conditions, and can be used to guide treatment decisions and interventions (35). For example, a machine learning model can generate a probability for each patient based on their characteristics. We observed that more severe trauma problems, such as higher ISS, rib fracture number, or intubation, increased the risk of pneumonia, consistent with clinical experience and previous data (5, 31). Consistent with previous studies, a more unstable physiological environment, e.g., lower pH value, systolic blood pressure, and haemoglobin, also increased the risk of pneumonia (39, 40, 43). As a result, the interpretable machine learning model built demonstrates good prediction results.

The study may have several limitations. First, it was a single-centre study with a limited sample size. Given that only four percent of patients with rib fractures develop flail chest, the sample size of flail chest patients in one medical centre was small (1). To mitigate the small sample size, we performed cross-validation (*k* = 3) of every model. However, selection bias was inevitable, and the model's efficacy for predicting pneumonia should be verified in future studies with larger sample sizes. Second, our study was conducted retrospectively. We can only suggest associated and correlated factors, not identify the main factors contributing to pneumonia. Furthermore, there is a possibility that our findings could be biased due to the long duration of patient enrolment. Third, whereas the missing numerical variables were imputed with weighted k-nearest neighbours, essential variables such as CRP and PH value had some missing values. Additionally, the accuracy may be improved by adding more variables currently not collected, such as comorbidity (40) (e.g., COPD, type 2 diabetes mellitus) and other predictors, such as smoking, alcohol usage, aspiration, and prehospital measures (44, 45). Fourth, we used variables within 48 h as independent variables instead of time-varying variables to predict the occurrence of pneumonia that cannot reflect the dynamic changes in of hemodynamic and metabolic variables in trauma patients. Finally, further external validation is needed. Fortunately, our team will continue to collect data on a prospective multicentre cohort study to refine the model.

## Conclusion

We successfully established seven ML models to predict the risk of pneumonia during hospitalization in flail chest patients. The XGBoost model has shown better performance than the LR, RF, SVM, ANN, GBDT, and NB models. We anticipate that it is a convenient risk stratification tool that clinicians can use to identify individualized treatment options for patients with multiple rib fractures. To our knowledge, this is the first machine learning based study to provide a novel approach for predicting pneumonia in flail chest patients. However, further external validation is required to test the generalization of our model.

## Data Availability

The raw data supporting the conclusions of this article will be made available by the authors, without undue reservation.
