# Supplementary material for: Predicting pneumonia during hospitalization in flail chest patients using machine learning approaches
Source: Front Surg. 2023 Jan 6;9:1060691. doi: 10.3389/fsurg.2022.1060691 (PMC9852626; doi:10.3389/fsurg.2022.1060691)
Supplement: Supplementary file 1 [file Datasheet1.docx]

Supplementary Material

# Supplementary Tables

**Supplementary table 1**: The baseline characteristics of mis-predicted patient.

|  | False-negative | | | False-positive | | |
| --- | --- | --- | --- | --- | --- | --- |
|  | patient A | patient B | patient C | patient A | patient B | patient C |
| Age(years) | 51 | 53 | 53 | 18 | 74 | 41 |
| Injure mechanism |  |  |  |  |  |  |
| Pedestrian accident | + | + | + | - | - | - |
| Traffic accident | - | - | - | + | - | + |
| Fall | - | - | - | - | + | - |
| Rib location |  |  |  |  |  |  |
| Left | + | + | + | + | - | - |
| Bilateral | - | - | - | - | + | + |
| Rib fracture number,n | 9 | 8 | 8 | 4 | 7 | 10 |
| ISS | 29 | 25 | 25 | 50 | 25 | 24 |
| Other AIS ≥ 3 | + | + | + | + |  |  |
| GCS | 14 | 15 | 15 | 6 | 15 | 15 |
| SBP (mm Hg) | 150 | 120 | 120 | 114 | 108 | 79 |
| DBP (mm Hg) | 94 | 71 | 71 | 70 | 68 | 51 |
| PH | 7.4 | 7.43 | 7.43 | 7.46 | 7.41 | 7.32 |
| Haemoglobin(g/L) | 128 | 141 | 141 | 65 | 90 | 76 |
| Tracheotomy | + | - | - | - | - | - |
| Blood transfusion | - | - | - | + | + | + |
| Emergency operative | - | + | + | + | - | - |
| Emergency in ICU | + | - | - | + | - | + |

'+' means yes, '-' means no. ISS, Injury Severity Score; Other AIS, abbreviated injury scale except for chest region; GCS, Glasgow Coma Scale; SBP, systolic blood pressure, DBP, diastolic blood pressure.

## Supplementary Figures


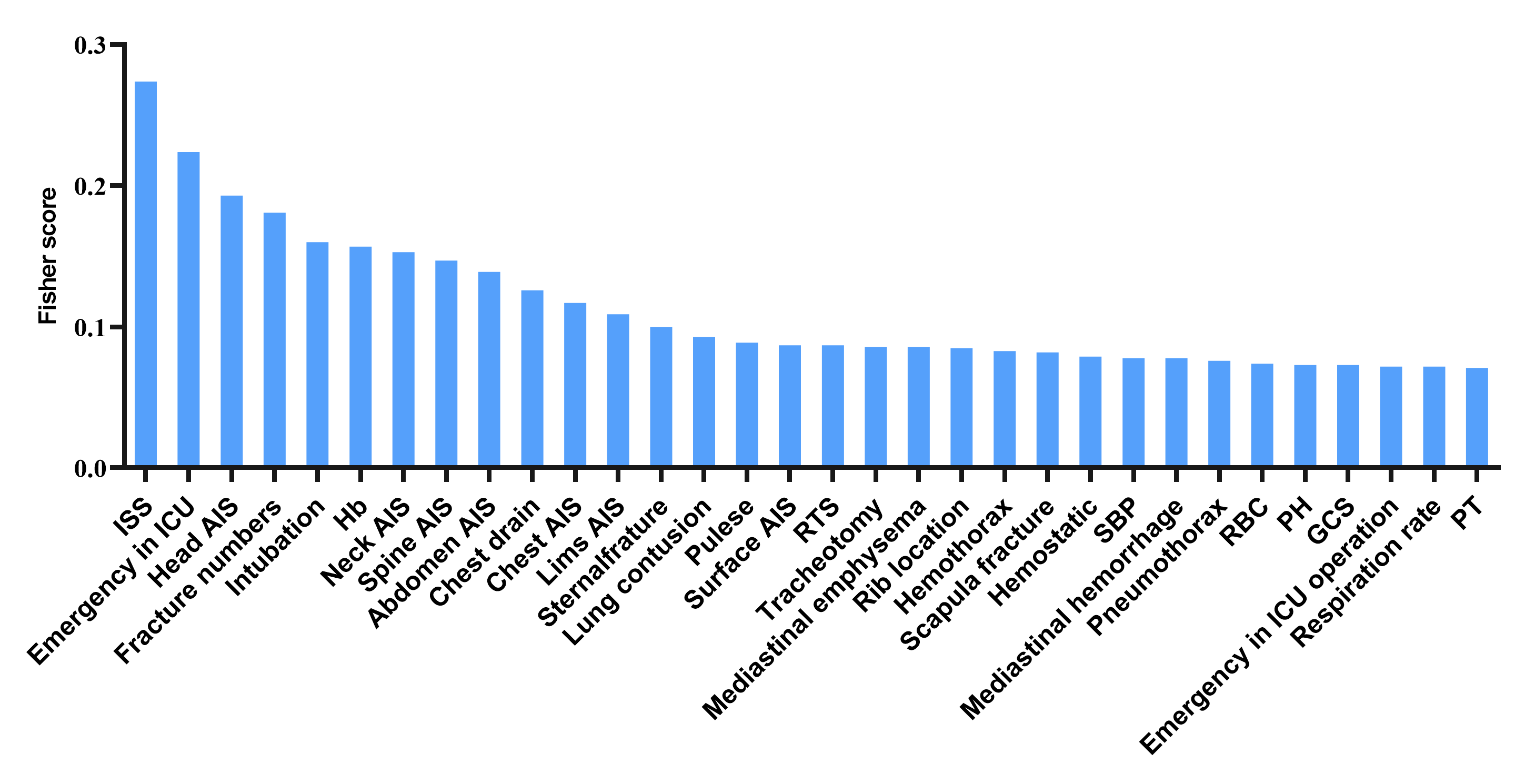


**Supplementary Figure 1.** Ranking of fisher scores for 32 features.
